# PFAS and Microplastics: Are Biodegradable Microplastics Less Harmful to the Environment?

**DOI:** 10.3390/molecules31142416

**Published:** 2026-07-09

**Authors:** Sonia Gaaied, Leilei Zhang, Terenzio Bertuzzi, Lucrezia Lamastra, Pier Paolo Becchi, Maria Grimaldi, Duccio Gallichi-Nottiani, Daniel Milanese, Corrado Sciancalepore, Nicoleta Alina Suciu

**Affiliations:** 1 Department for Sustainable Food Process (DiSTAS), Faculty of Agriculture, Food and Environmental Sciences, Università Cattolica del Sacro Cuore, 29121 Piacenza, Italy; leilei.zhang@unicatt.it (L.Z.); lucrezia.lamastra@unicatt.it (L.L.); pierpaolo.becchi@unicatt.it (P.P.B.); 2 Department of Animal Science, Food and Nutrition-DIANA, Università Cattolica del Sacro Cuore, Via Emilia Parmense 84, 29122 Piacenza, Italy; terenzio.bertuzzi@unicatt.it; 3 Department of Engineering for Industrial Systems and Technologies, Parco Area delle Scienze, 43124 Parma, Italy; maria.grimaldi@unipr.it (M.G.); duccio.gallichinottiani@unipr.it (D.G.-N.); daniel.milanese@unipr.it (D.M.); corrado.sciancalepore@unipr.it (C.S.)

**Keywords:** PFAS, adsorption, biodegradable microplastics

## Abstract

The monitoring of environmental pollution has attracted growing attention to per- and polyfluoroalkyl substances (PFASs) due to their long persistence and resistance to natural degradation. Despite extensive research into the occurrence of PFASs in the environment, their interactions with biodegradable polymers remain understudied, highlighting a substantial knowledge gap regarding their adsorption capacity and associated toxicological implications. Therefore, the main objective of the present study was to evaluate PFAS adsorption onto biodegradable microplastics (BMPs) and compare their behavior to non- BMPs. Although biodegradable plastics (BPs) are generally considered environmentally friendly polymers, the present study indicates that they can act as vectors for PFASs as shown by LC-MS-MS analysis. Based on the current results, PFAS adsorption depends on the polymer type and PFAS chemical structure. Although BMPs are not the only carriers of PFASs, compared with various natural particles, their persistence and mobility can still influence the transport and bioavailability of PFASs in the environment. Future studies are required for assessing the environmental fate of PFASs in systems containing biodegradable polymer, in order to enhance predictive accuracy and mitigate associated risks to both the environment and human health.

## 1. Introduction

Per- and polyfluoroalkyl substances (PFASs) are a class of persistent organic pollutants that are widespread in the environment and resistant to degradation, raising significant ecological and health concerns [1,2,3,4]. In the environment, PFAS pollution frequently co-occurs with other emerging contaminants, particularly microplastics (MPs). Although their individual toxicity has been extensively studied [5,6,7,8], PFAS-MP interactions and combined behavior remain poorly characterized [9]. Therefore, the relevant literature published between 2018 and 2026 was screened using the ‘Rayyan Systematic Review’, enabling a comprehensive synthesis of current knowledge on PFAS-MP interactions. A total of 69 references were identified from the Scopus (58), Web of Science (WoS, 49), and PubMed databases (30). The most frequently studied polymers were polystyrene (PS), followed by polyethylene (PE) and polyvinyl chloride (PVC). The PFAS class was mentioned in 47 publications. Perfluorooctanoic acid (PFOA) was referenced most frequently in the literature, followed by sodium perfluorooctanesulfonate (PFOS) and perfluorobutanoic acid (PFBA). Eighteen publications focused on water as the environmental matrix, while soil was mentioned in five studies. Forty-seven studies considered the adsorption of PFASs in microplastics/nanoplastics, while 27 publications reported on toxic effects in water, soil organisms, and humans.

Wang et al. [10] were the first to report the sorption behavior of perfluorooctanesulfonic acid (PFOS) and perfluorooctanesulfonamide (FOSA) on three types of conventional MPs (CMPs): polyethylene (PE), polystyrene (PS) and polyvinyl chloride (PVC). Subsequent studies have focused on the presence of PFASs on CMPs, their sorption mechanisms, the factors influencing sorption behavior and their possible toxicity to terrestrial organisms and humans [11,12,13]. In the literature, the interaction between PFASs and CMPs may occur in aquatic and soil environments. The mechanisms involved in sorption capacity have been described as follows. PFAS sorption by non-BMPs is mainly governed by electrostatic and hydrophobic forces, along with hydrogens bonds, PFAS structure and environmental factors (including pH, organic matter, ionic strength, and temperature) [10,11,13]. Based on previous research, CMPs have the potential to adsorb PFASs affecting their distribution and transport in the environment [9,12]. However, their effects under environmentally realistic conditions remain unclear. Moreover, research on factors that affect the desorption and bioavailability of PFASs by CMPs, as well as biodegradable MPs’ (BMPs’) ability to adsorb PFASs needs further investigation.

Biodegradables plastics (BPs) are derived from petrochemical and bio-based sources and used worldwide as alternatives to conventional plastics (CPs) [14]. They include polymers such as adipate-co-terephthalate (PBAT), polylactic acid (PLA) and poly(3-hydroxybutyrate-co-3-hydroxyhexanoate) (PHBH), which are considered a more environmentally friendly alternative to conventional, non-biodegradable, petroleum-based plastics [15,16]. To improve their suitability for packaging, diverse bio-based additives such as inulin, orange peel and pasta by-product can be incorporated [17]. Global bioplastic production is estimated to reach 7.43 million tons by 2028, reflecting the growing demand for sustainable alternatives, while CPs still account for 90% of total production [18,19,20]. Although bioplastics and plant-derived materials are promoted as environmentally friendly compared to traditional plastics, it is uncertain whether they can be a promising solution to waste disposal and global plastic pollution [21]. Biodegradable polymers may still pose ecological risks, including incomplete biodegradation, release of additives and vectors of contaminants [22]. Thus, they may cause toxicity to living organisms in a similar manner as CMPs [22,23]. In addition, BMPs can adsorb pollutants more than CMPs, such as phenanthrene binding to PBAT compared with PE and PS [24], raising concerns about their role in transporting contaminants and their overall ecological impact. Particularly, their interaction with persistent pollutants such as PFASs highlights the need to better understand their ecotoxicological implications. Currently, only two studies have investigated PFAS adsorption onto BMPs in comparison with non-biodegradable polymers [25,26], and information regarding both pure BMPs or blend BMPs remain limited. Ateia et al. [26] examined PFAS sorption on both conventional polymers and biodegradable polylactic acid (PLA), reporting a relatively high affinity of PFASs for PLA compared to several CMPs. More recently, Tumrani et al. [25] demonstrated that aging significantly enhances PFAS adsorption onto PLA due to surface oxidation and the formation of oxygen-containing functional groups, which strengthen electrostatic interactions and hydrogen bonding.

Current research lacks a comprehensive assessment of interactions between PFASs and BPs, underscoring a substantial knowledge gap regarding BPs’ sorption behavior and potential to act as carriers of PFASs [27].

Therefore, the main objective of the present study was to investigate the interaction of BMPs with PFASs to improve understanding of their role as contaminant carriers in the natural environment. Specifically, the study aimed to: (i) evaluate the PFAS adsorption potential of BMPs (PBAT, PLA, and PHBH) either pure or blended; and (ii) compare PFAS sorption behavior of BMPs and CMPs to assess the influence of polymer composition.

## 2. Results

### 2.1. Adsorption Behavior of PFASs onto PBAT-P and PBAT-I 10%

**Figure 1:**
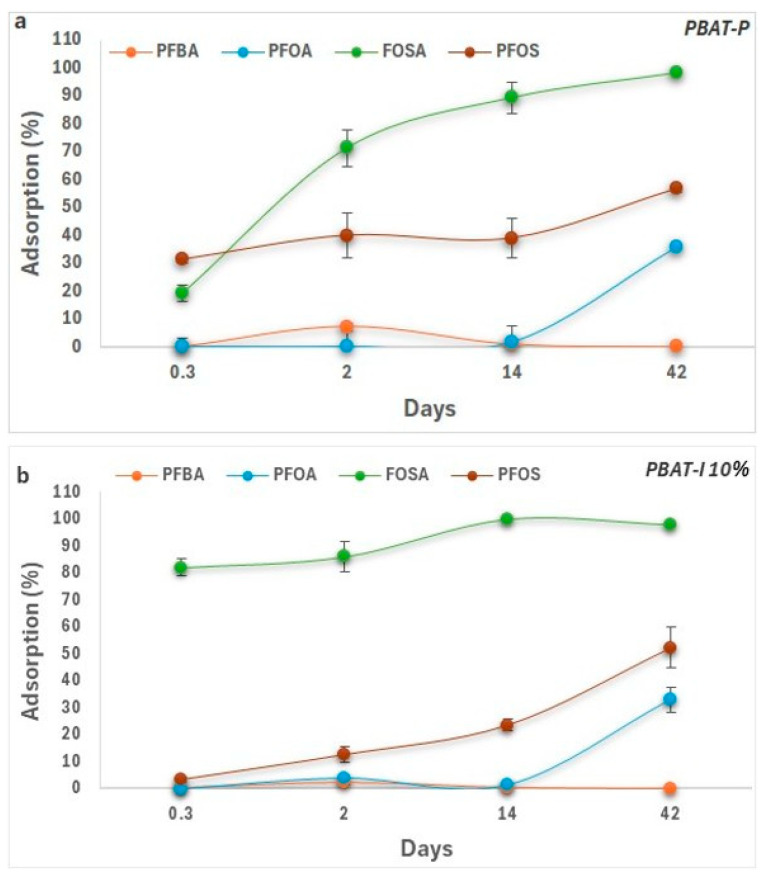
Adsorption percentages of PFASs onto (**a**) PBAT-P and (**b**) PBAT-I 10% in water.

Figure 1 presents the percentages of PFAS adsorption onto PBAT-P (a) and PBAT-I 10% (b). For both BMPs significant differences were observed among all PFASs depending on the day (Table S2, *p* < 0.05). The sulfonates FOSA and PFOS showed the highest adsorption. In contrast, the carboxylic acids PFOA and PFBA showed low to negligible adsorption percentages, indicating the influence of carbon chain length and functional group on PBAT adsorption behavior. Overall, FOSA, PFOS and PFOA exhibited time-dependent sorption behavior, with statistically significant differences at 42 days for both polymer types (Table S2, *p* < 0.05). The Kd values (Table 1), based on concentrations over 42 days, show the same trend: higher values for longer-chain compounds. Regarding the inulin effect, FOSA showed significant affinity over time, while PFOA affinity enhanced significantly as early as on day 2 (Table S3, *p* < 0.05).

### 2.2. Adsorption Behavior of PFASs onto PLA-P and PLA-OPP 5%

**Figure 2:**
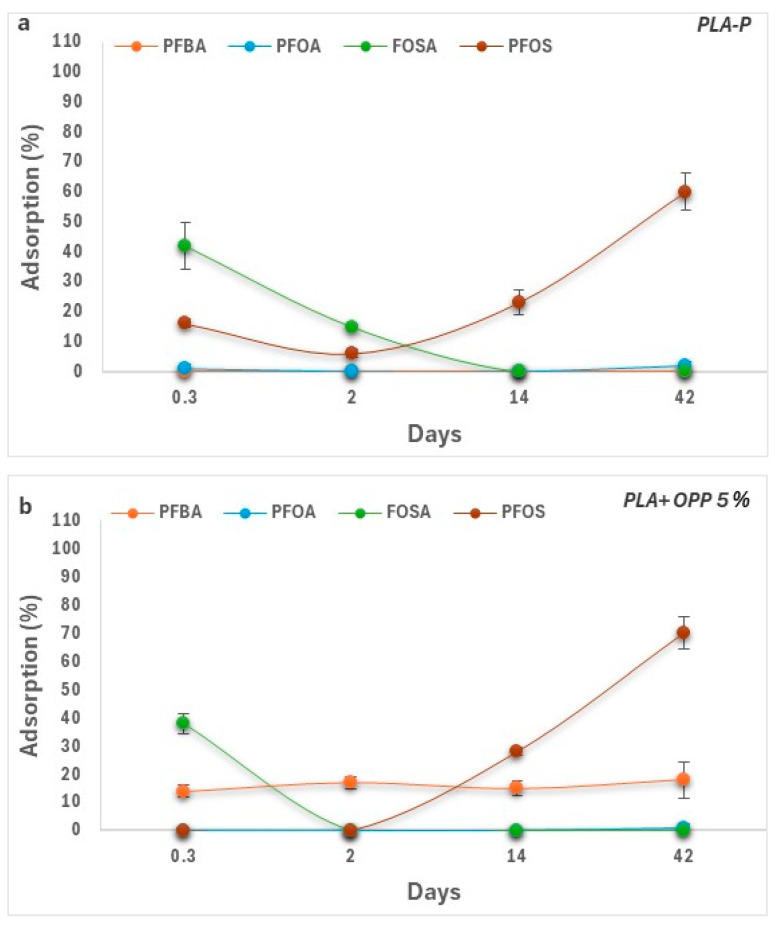
Adsorption percentages of PFASs onto (**a**) PLA-P and (**b**) PLA-OPP 5% in water.

Figure 2 presents the percentages of PFAS adsorption onto PLA-P (a) and PLA-OPP 5% (b). PLA-P showed no measurable affinity for PFBA and PFOA over time. In contrast, PFOS adsorption increased significantly over time and picked up at 42 days suggesting a stronger affinity for longer-chain sulfonate (Table S2, *p* < 0.05). FOSA was significantly adsorbed within 2 days and turned back into solution within 14 days (Table S2, *p* < 0.05). Regarding PLA-OPP 5%, the adsorption percentage of PFBA showed a slight increase and remained stable over time and showed significant differences regarding other PFASs (Table S2, *p* < 0.05). PFOS adsorption reached 70% in 42 days and demonstrated time-dependent sorption behavior. In comparison, PFOA remained mostly not adsorbed, whereas FOSA was adsorbed within 8 h and desorbed over time (Table S2, *p* < 0.05). The Kd values (Table 1), based on 42 days of adsorption, show higher values for the sulfonate PFOS. For the carboxylic acids PFOA and PFBA no equilibrium was reached. The incorporation of 5% OPP into PLA enhanced its affinity for PFBA, resulting in significant adsorption compared to pure PLA (Table S3, *p* < 0.05).

**Table 1:** Distribution coefficient Kd (mL/g) at 42 days.

		Kd (mL/g)					
Functional Group/C-n	Acronyms	PBAT Pure	PBAT I-10%	PLA Pure	PLA OPP-5%	PHBH Pure	PHBH PR-10%
Carboxylic acids (4 C)	PFBA	41.2	n.eq	n.eq	43.9	n.eq	10.53
Carboxylic acids (8 C)	PFOA	111.45	98	4.08	2.02	n.eq	6.19
Sulfonates (8 C)	PFOS	259.24	218.94	300	466.67	56.41	1338.46
Sulfonamide (8 C)	FOSA	9800	9800	n.eq	n.eq	n.eq	n.eq

### 2.3. Adsorption Percentages of PFASs onto PHBH-P and PHBH-PR 10%

**Figure 3:**
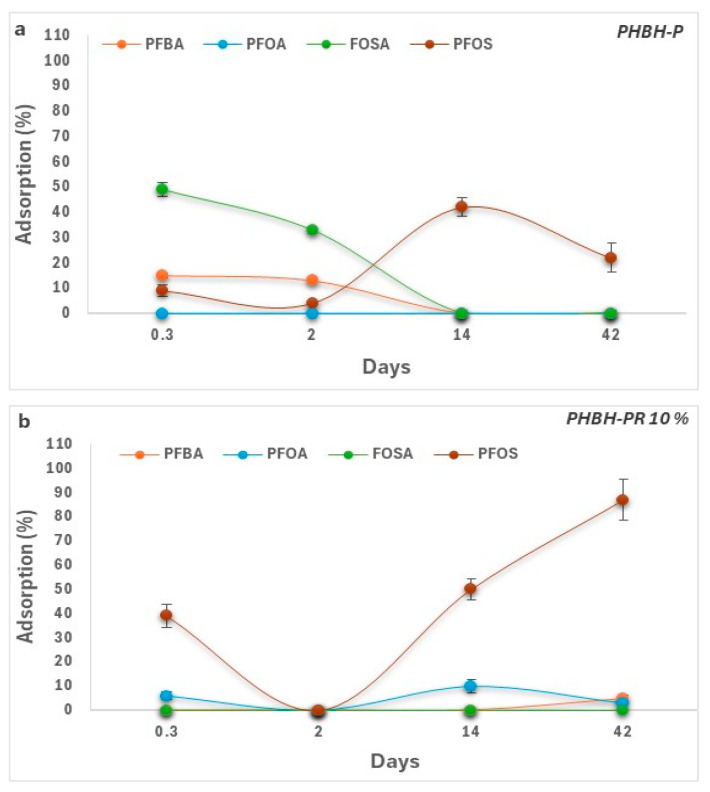
Adsorption percentages of PFASs onto (**a**) PHBH-P and (**b**) PHBH-PR 10% in water.

Both PHBH-P and PHBH-PR 10% demonstrated significant affinity for adsorbing PFOS, with an enhanced percentage in the composite material (Figure 3a,b, Tables S1 and S2; *p* < 0.05). PFOS adsorption exhibited a dynamic sorption pattern characterized by alternating increases and decreases over time, with a statistical difference to other PFASs from 14 days (Table S1; *p* < 0.05). Pure PHBH showed no adsorption of PFOA, whereas PFBA was slightly adsorbed within 2 days (Table S2, *p* < 0.05) and turned back into solution within 14 days. FOSA showed similar behavior to PLA (pure and blended), with moderate significant adsorption within 2 days in pure PHBH (Table S2, *p* < 0.05) and no adsorption on PHBH-PR 10%. Moreover PHBH-PR 10% showed a slight increase in PFOA adsorption characterized by oscillating reversible adsorption (Tables S1 and S2; *p* < 0.05). Consistent with these observations, the Kd value (Table 1) at 42 days was higher for the sulfonate PFOS with PHBH reflecting its strong binding affinity. For the carboxylic acid PFOA and PFBA, no equilibrium was reached, indicating negligible interaction.

### 2.4. Comparative Adsorption of Each PFAS Across All BMPs Under Same Day

Overall, significant differences in adsorption were observed when comparing the same PFAS across different tested BMPs at the same time point (Table S3, *p* < 0.01). Regarding PFBA, PLA-5% OPP showed significantly higher adsorption than the other BMPs at all days, while PHBH-P showed significantly higher adsorption at 0.3 and 2 days. For PFOA, PBAT-I demonstrated significantly higher adsorption at 2 and 42 days, whereas PHBH-10% showed significantly higher differences at 0.3 and 14 days. PFOS exhibited significantly higher differences at 0.3 and 2 days for PBAT-P, while PHBH + 10% showed significantly higher adsorption at all time points except day 2. For FOSA, PBAT-I demonstrated significantly higher adsorption across time compared to the other BMPs.

## 3. Discussion

PFAS adsorption onto BPs has become an emerging area of research due to their biodegradability, which may enhance the release and environmental spread of PFASs through desorption processes. Previous research on MPs-PFAS interactions primarily focused on CPs (PE, PVC and PS) [10,11,26,28,29]. However, with the increased use of BPs, it is important to investigate their role in PFAS behavior, particularly their adsorption and transport, to prevent long-term environmental consequences.

In this study, the adsorption of four PFASs was assessed through three types of BMPs (pure or blended) for 42 days.

PBAT is a synthetic biodegradable aliphatic–aromatic copolyester with mechanical properties resembling low-density polyethylene (LDPE) widely used in packaging and agricultural film [30]. Due to its limited mechanical properties, PBAT-based composites and blends have been manufactured to improve their quality [14,31]. Regarding PFAS adsorption on PBAT-P and PBAT-I 10, FOSA exhibited the highest adsorption followed by PFOS and PFOA, while PFBA was not adsorbed. Furthermore, the incorporation of inulin fillers increased PFOS adsorption. In fact, PFAS adsorption is significantly influenced by hydrophobic interactions, particularly with hydrophobic adsorbents such as PBAT [32]. This indicates that the aliphatic part of the molecules plays a predominant role in their partitioning, as has been reported for other groups of organic compounds, as well as hydrocarbons [33]. Llorca et al. [11] observed similar behavior, with sulfonamide PFASs exhibiting higher adsorption onto PS than PFAS carboxylate or sulfonate.

PLA, a bio-based and biodegradable thermoplastic aliphatic polyester derived from natural resources, was also evaluated for its potential to adsorb PFASs. PLA is considered suitable for substituting CPs and can be blended with natural filler to improve its performance in the market [34]. Regarding the adsorption percentages of PFASs to PLA-P and PLA-OPP 5%, it was observed that PFOS was the most adsorbed. In contrast, PFOA and PFBA showed low-to-no adsorption. These findings illustrate that the sulfonate PFOS (8C) has a strong affinity to PLA compared to PFAS carboxylic acids. Moreover, the incorporation of orange peel powder induced a slight increase in PFBA adsorption. This can be explained by the fact that the adsorption characteristics on PLA-OPP 5% are affected by surface chemistry rather than PFAS carbon chain length. Adding orange peel as a quality-enhancing additive may introduce more functional groups that improve PFBA adsorption. These findings are consistent with previous studies where they reported that the adsorption mechanism to PLA is primarily governed by hydrophobic interactions, as PFAS molecules tend to adhere to non-polar surfaces [35]. Moreover, incorporating functional groups or other materials such as calixarenes can improve PLA adsorption properties by increasing hydrophilicity and surface area [34]. According to Ateia et al. [26], hydrophobic interactions are the main factor in PLA adsorption to PFASs, with adsorption affinity listed as PFOS > PFOA > GenX. However, research has also reported that unmodified polymers had lower sorption compared to real MPs, including PLA variants, due to roughness surface difference as well as the presence of fillers [27], as observed for PFBA adsorption on blended PLA in this study. In this regard, a previous study showed that the adsorption of undecafluorohexanoic acid (UFHA), a C6, on amidine-functionalized polystyrene demonstrates that even short-chain PFASs can bind to charged MPs in the presence of electrostatic interaction and hydrogen bonding [36]. Another factor that may influence PFAS adsorption into BMPs is aging. Tumrani et al. [25] showed that pristine PLA MPs exhibited low PFOA physisorption, whereas PLA aged for 80 days showed significantly higher adsorption capacity dominated by chemisorption via stronger electrostatic interactions and hydrogen bonding.

PHBH is a neutral hydrophobic bio-based copolymer that degrades naturally and research about its adsorption on PFASs is still ongoing. In this study, PFOS was preferentially adsorbed by pure PHBH, while PFBA and PFOA remained mostly in water. The insertion of pasta by-products as a filler to PHBH enhanced PFOS adsorption and slightly increased PFOA sorption while PFBA remained unabsorbed. PHBH is composed of 3-hydroxybutyrate (3HB) and 3-hydroxyhexanoate (3HHx) [37]. Structurally, PHBH contains carbonyl groups and hydrophobic alkyl [38]. Consequently, adsorption is likely to occur through hydrophobic interactions instead of electrostatic binding [39,40]. PFAS affinity for PFOS compared to PFOA under similar conditions suggests that head group chemistry plays a main role [39]. In particular, the sulfonate group of PFOS exhibits distinct interfacial behavior and better surface activity relative to the carboxylic group promoting its adsorption onto hydrophobic polymers [39,40]. Current evidence indicates that pure PHBH would act similarly to other unmodified hydrophobic polymers, adsorbing more hydrophobic long-chain ones rather than shorter ones.

Overall, across the tested BMPs, PFAS affinity generally increased for long-chain sulfonates (PFOS), which showed high and persistent adsorption. Long-chain sulfonamides (FOSA) demonstrated unstable affinity, and time-dependent and often reversible adsorption. In contrast, short-chain carboxylic acids (PFBA and PFOA) showed minimal-to-negligible adsorption.

Regarding the filler effect, inulin, orange peel powder, and pasta residues can modify the surface chemistry and interfacial properties of polymer composites. These bio-based additives may introduce functional groups (–OH, –COOH, C=O) that enhance adsorption performance and shift the hydrophilic–hydrophobic balance, while the plastic provides structural support and helps distribute active sites [41,42]. Bio-based fillers may also increase surface roughness and porosity due to their fibrous, heterogeneous nature, creating additional adsorption sites [43] which together may influence PFAS adsorption affinity.

An additional observation from this study is the reversible adsorption–desorption behavior of PFOS and PFOA with most tested BMPs. This change in equilibrium may reflect partial release after adsorption supporting the absence of a linear sorption. Consistent with our findings, a recent study showed that PFOS interacts dynamically with environmentally collected MPs, indicating reversible adsorption [44]. Similarly, Salawu et al. [45] reported that PFAS adsorption onto secondary MPs is a spontaneous process characterized by fluctuating adsorption patterns across time, governed primarily by hydrophobic interactions, with electrostatic forces and hydrogen bonding. Therefore, the adsorption–desorption patterns observed for the tested BMPs may reflect the dynamic and reversible nature of PFAS sorption processes. Such variability may also be influenced by non-equilibrium conditions, surface heterogeneity of MPs and polymer aging [46]. Notably, the reversible adsorption–desorption behavior observed in this study suggests that BMPs may function both as contaminant sinks and as secondary contaminant sources depending on environmental conditions.

Taken together, the results suggest that the adsorption of PFASs onto BMPs is likely controlled by several mechanisms. In addition to hydrophobic interactions, electrostatic forces, and hydrogen bonding, PFAS adsorption onto MPs can also be governed by van der Waals interactions and π–π stacking, while being influenced by polymer type, PFAS properties, and environmental conditions [29,47].

When compared with CMPs, BMPs do not inherently represent less hazardous alternatives in the context of PFAS contamination. BMPs can exhibit similar or even higher sorption efficiencies of PFASs than traditional plastic [22,48,49]. This behavior is generally attributed to their higher oxygen content and greater surface polarity as well as rubbery domains, which increase their interaction with polar substances like PFASs under specific conditions. Similar behavior was also demonstrated for other contaminants, where biodegradable PBAT and PBS/PLA adsorb more than PE, PS and PP, especially for hydrophobic chemicals [24,50]. However, CMPs still exhibit significant PFAS sorption, as their sorption capacity can increase significantly following environmental aging. In fact, a study on polycyclic aromatic hydrocarbons (PAH) adsorption showed lower affinity to PLA than other non-BPs thus indicating that not all BPs behave the same way [51]. Thus, PFAS-MP interactions depend on physicochemical properties of polymers and environmental conditions, rather than being determined by polymer biodegradability only [52].

In aquatic or terrestrial environments, BPs undergo fragmentation, generating smaller particles that can interact with PFASs [17,53,54]. Both BMPs and CMPs can act as “Trojan horses”, facilitating the transport, bioavailability, and trophic transfer of PFASs and other pollutants in environmental and biological systems [13,55,56], thereby inducing ecotoxicological effects [54,57]. From a toxicological perspective, adsorption does not necessarily imply increased biological effects, since desorption depends on several factors.

Our results suggest that BMPs may act as potential PFAS carriers under controlled laboratory conditions. Such conditions do not fully reproduce natural environments, where salinity, dissolved organic matter, pH variations, mineral particles, temperature fluctuations, and microbial colonization may influence PFAS adsorption [58,59]. Therefore, further investigations under naturally realistic systems are necessary to better assess their environmental relevance and ecological implications.

## 4. Materials and Methods

### 4.1. Reagents and Standards

HPLC-grade methanol, acetonitrile, water and ammonium acetate were purchased from Carlo Erba Reagents S.r.l. (Milan, Italy), while the PFOS standard was supplied by Wellington Laboratories (Guelph, ON, Canada), and the PFOA (perfluorooctanoic acid), PFBA (perfluorobutanoic acid) and FOSA standards were supplied by Merck Life Science S.r.l. (Milan, Italy). The molecular formulas of the PFASs are summarized in Table S1 of the Supplementary Materials. Individual stock solutions were prepared in methanol and stored at −18 °C: 9.8 g/L for PFBA, 10 g/L for PFOA and FOSA, and 6 mg/L for PFOS. Working solutions ranging from 1 to 5 mg/L were prepared in water whereas calibration curve solutions ranging from 0.05 to 50 µg/L were prepared in a water/acetonitrile mixture (40% *v*/*v*).

### 4.2. Biodegradable Microplastic Preparation

#### 4.2.1. PBAT Pure and PBAT-Inulin Polymers

The purchased PBAT in this study is ECOWORLD (Jinhui ZhaoLong High Technology Co., Ltd., Lvliang, China). PBAT is a fully biodegradable and compostable polymer, designed for flexible film extrusion, injection molding, and packaging. Its M_W_ is approximately 106 kDa. Commercial PBAT granules were milled in cryogenic conditions to obtain a polymer powder, which was then dried overnight in a static oven at 40 °C. The inulin was sieved to an average particle size of between 50 and 150 µm, and then dried overnight at 40 °C. The mixture of powders at different concentrations (5–10% *w*/*w*) was then processed using twin-screw extrusion to produce composite materials. After extrusion, the pure PBAT (PBAT-P) and composite material (PBAT-I 10%) were milled and sieved under 150 µm after cooling with liquid nitrogen.

#### 4.2.2. PLA Pure and PLA Orange Peel Powder Polymers

The selected PLA is Luminy L105 (Total CorbionPLA DV, Gorinchem, The Netherlands), a semi-crystalline polymer of the L-PLA (PLLA) type. It is a stereochemically pure polylactic acid with a high content of the L-isomer (greater than 99% L-isomer). Its weight-average molecular weight (M_W_) is 75–90 kDa. The degree of crystallinity achievable with PLA Luminy L105 (in its pure form or after appropriate annealing) ranges from 40% to 45%. However, the final degree of crystallinity is strongly influenced by the material’s thermal history and processing conditions, with values below 15% typically observed after twin-screw extrusion. The composite made of pure PLA (PLA-P) was selected as a polymer to be filled with orange peel powder (OPP). The OOP was obtained from the by-products from orange juice production provided by a company from Southern Italy. The orange peels were received as dried powder and characterized for moisture content and stabilized prior to compounding. PLA-OPP composites with filler contents of 5 percent (PLA-5% OPP) were prepared by twin-screw extrusion and subsequently processed for thermo-mechanical characterization and film production.

#### 4.2.3. PHBH Pure and PHBH Pasta Fillers Polymers

The polyhydroxybutyrate-co-hexanoate (PHBH) used is IamNATURE (MAIP srl, Turin, Italy), with the M_W_ ranging from 400 to 450 kDa. PHBH is a 100% naturally bio-based polymer: it is synthesized through the bacterial fermentation of renewable carbon sources (sugars, vegetable oils, or agri-food waste). PHBH was selected as the polyhydroxyalkanoate (PHA) polymer matrix to be compounded with regrind pasta as filler. Regrind pasta, provided by an Italian producer, was heat-treated to stabilize the powder for compounding. To reduce the humidity content, the powder was treated under vacuum at 180 °C for 48 h. FT-IR characterization was performed to determine the presence of humidity within the powder. The powder was sieved to estimate the average particle size distribution and the portion of powder below 50 µm was employed for the composite production. After these preparation steps, PHA-based composites containing pasta fillers (PHBH + 10% PR) were prepared by twin-screw extrusion.

### 4.3. Adsorption Study

A series of experiments has been conducted to investigate the sorption capacity of diverse BMPs for PFAS compounds. Following the protocol developed by Wang et al. [10], all experiments were carried out in 15 mL polypropylene copolymer (PPCO) Nalgene centrifuge tubes (Rochester, NY, USA), containing 0.05 g of PBAT-P and PBAT-I, PLA-P and PLA-5% OPP, PHBH-P and PHBH + 10% PR (size range: 100–200 µm) in 10 mL of PFOA, PFOS, FOSA, or PFBA at a concentration of 50 µg/L. The tubes were shaken at 150 rpm and kept at 25 °C for 8 h, 2, 14 and 42 days. Procedural blanks containing PFASs were prepared under the same testing conditions to determine possible PFAS loss or sorption to the tubes. The samples were then centrifuged at 4500 rpm for 5 min and filtered through a 0.22 µm filter (RC, VWR International S.r.l., Milan, Italy). Finally, the filtered supernatants were transferred into an HPLC vial for LC-MS/MS analysis.

As proposed by Llorca et al. [11], the adsorption percentages were calculated as the mean ± SD (n = 2) using Equation (1)

(1) $$\%Ads=100-\frac{A_{t}}{A_{0}}\times 100$$

where %Ads is the percentage of adsorption. A_t_ is the concentration of compound A remaining in the solution after time t and A_0_ is the initial concentration of compound A. The partition/distribution coefficient (Kd) was calculated in accordance with Equation (2) based on OECD guideline 106 (OECD_106):

(2) Kd=%Adseq100−%Adseq × V0msorbent(mLg)

where %Ad_seq_ is the percentage of adsorption at equilibrium, V_0_ is the initial volume of the aqueous phase and m_sorbent_ is the mass of the corresponding BMPs. According to Equation (2), the K_d_ of each compound is directly proportional to its tendency to be adsorbed onto a PBAT-P, PBAT-I 10%, PLA-P, PLA-OPP 5%, PHBH-P and PHBH-PR 10% in water. Additionally, Equation (2) assumes negligible loss of compounds.

### 4.4. LC-MS/MS Analysis

The system comprised a Vanquish pump and autosampler, as well as a TSQ Fortis triple-quadrupole mass spectrometer (Thermo Fisher Scientific, San Jose, CA, USA). Separation was performed using an X-Select HSS T3 Column (100 Å, 3.5 μm, 2.1 mm × 150 mm; Waters, Milford, MA, USA). The injection volume was 10 µL, the indicated run time was 15 min, and the flow rate was 0.2 mL/minute. The mobile phases were ultra-pure water containing 2 mM ammonium acetate (phase A) and acetonitrile containing 2 mM ammonium acetate (phase B). The gradient of solvent B was set up as follows: 0–1 min at 40%; 4–9 min from 40% to 95%; 9–10 min at 40%; and re-equilibration for 7 min. The ionization was carried out with an H-ESI interface (Thermo-Fisher) in negative mode as follows: spray capillary voltage 2.5 kV, sheath and auxiliary gas 35 and 10 psi, respectively, vaporizer and ion transfer tube temperature were 200, 275, 400 and 350 °C for HFBA, PFOA, PFOSA and PFOS, respectively. Fragmentation parameters, parent and fragmentation ions, selected through direct infusion of standard solutions in the mass spectrometer, are reported in Table 2. The limits of detection (LOD) and of quantification (LOQ) were calculated using the calibration curve’s slope and the standard deviation of the response.

### 4.5. Statistical Analysis

All data are expressed as percentages (means of two replicates). The effects of the experimental factors on PFAS adsorption to BMPs were analyzed using a multivariate General Linear Model (GLM) using SPSS Statistics 30. Differences among means were assessed using Tukey’s post hoc test (Tables S2 and S3), and results were considered statistically significant at *p* < 0.05.

## 5. Conclusions

Even though BPs are generally considered as environmentally friendly polymers, studies have shown that they can be active vectors for PFASs. Based on the current results PFAS adsorption depends on the polymer type, and the PFAS chemical structure. Generally, sorption occurs through hydrophobic and fluorophobic interactions and it could be influenced by electrostatic interactions and hydrogen bonds [55]. The functional groups of PFASs have a considerable impact on the affinity, with sulfonates and sulfonamides having higher sorption than carboxylates [11]. Although MPs are not the only carriers of PFASs, compared with natural particles such as sediments or organic matter, their persistence and mobility can still influence the transport and bioavailability of PFASs in the environment [60]. Laboratory and field studies show that aging, biofilms and environmental solids can either suppress or enhance PFAS sorption [58]. However, key factors that drive PFAS adsorption into BMPs remain insufficiently investigated. Future studies are required to assess the environmental fate of PFASs from biodegradable polymers, with particular emphasis on elucidating adsorption mechanisms under environmentally relevant conditions, to mitigate potential risks to both environmental and human health.

## Figures and Tables

**Table 2:** MS-MS parameters and LOD/LOQ values.

	Parent Ion	Fragmentation Ion	Collision Gas (Ar)	Collision Energy	LOD	LOQ
HFBA	213.0	169.0	1.5	15	4.6	15.4
PFOA	413.1	369.1	1.5	13	0.3	1.0
PFOSA	498.1	78.1, 169.0, 218.9	2.5	46	0.4	1.4
PFOS	499.1	80.5, 99.5, 229.8	2.5	46	0.3	1.1

## Data Availability

The original contributions presented in this study are included in the article. Further inquiries can be directed to the corresponding authors.

## Supplementary Materials

The following supporting information can be downloaded at: https://www.mdpi.com/article/10.3390/molecules31142416/s1, Figure S1: Chemical structures of the biodegradable polymers used in this study: PBAT (A), PLA (B), and PHBH (C); Table S1: Chemical names, abbreviations, and molecular formulas of the PFAS investigated in this study; Table S2: Statistical comparisons of PFOS responses across exposure conditions. Different uppercase letters indicate significant differences among PFOS types within the same exposure time and the same BMPs (Tukey’s HSD test, *p* < 0.05). Different lowercase letters indicate significant differences among exposure time within the same PFAS type (Tukey’s HSD test, *p* < 0.05); Table S3: Statistical comparisons of PFAS adsorption across BMPs types at each exposure time. Different lowercase letters indicate significant differences among plastic types within the same PFAS and the same day (Tukey’s HSD test, *p* < 0.05).

## Abbreviations

The following abbreviations are used in this manuscript:

MPs	microplastics
PFAS	per- and polyfluoroalkyl substances
PFOS	sodium perfluorooctanesulfonate
FOSA	perfluorooctanesulfonamide
PFBA	perfluorobutanoic acid
PFOA	perfluorooctanoic acid
PE	polyethylene
PS	polystyrene
PVC	polyvinyl chloride
PBAT	polybutylene adipate-co-terephthalate
PLA	polylactic Acid
PHBH	poly(3-hydroxybutyrate-co-3-hydroxyhexanoate)
Kd	distribution coefficient (mL/g)
LDPE	low-density polyethylene
3HB	3-hydroxybutyrate
3HHx	3-hydroxyhexanoate
PAH	polycyclic aromatic hydrocarbons
