# Supplementary material for: PFAS and Microplastics: Are Biodegradable Microplastics Less Harmful to the Environment?
Source: Molecules. 2026 Jul 9;31(14):2416. doi: 10.3390/molecules31142416 (PMC13413754; doi:10.3390/molecules31142416)
Supplement: Supplementary file 1 [file molecules-31-02416-s001.zip › molecules-4322498-supplementary.pdf]

Supplementary materials

Table S1. Chemical names, abbreviations, and molecular formulas of the PFAS investigated in this study.

**Chemical name**	**Abbreviation**	**Molecular formula**
Perfluorooctanesulfonamide	PFOSA	C_8-H_2-F_{17}-N-O_2-S
Heptafluorobutyric acid	HFBA	$C_4HF_7O_2$
Perfluorooctanoic acid	PFOA	$C_8HF_{15}O_2$
Sodium perfluorooctanesulfonate	PFOS	$C_8F_{17}NaO_3S$

A) PBAT repeating unit

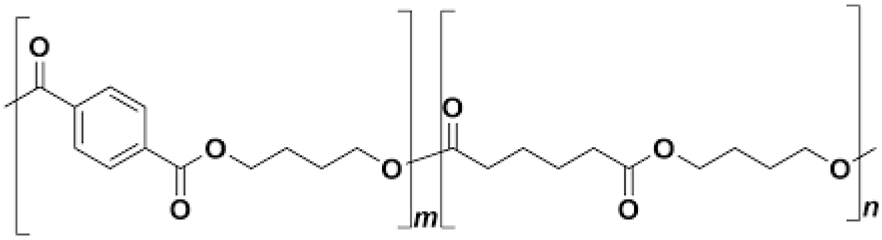

B) PLA repeating unit

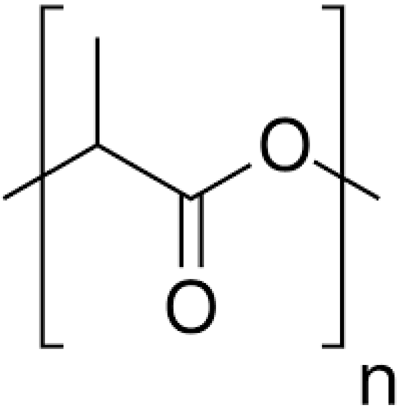

C) PHBH repeating unit

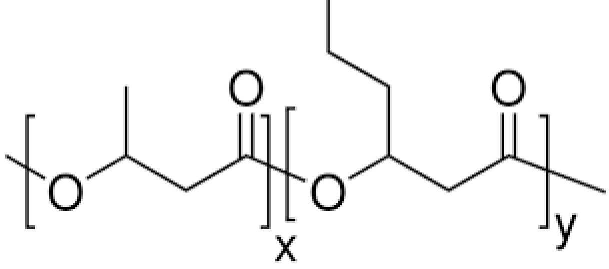

Figure S1. Chemical structures of the biodegradable polymers used in this study: PBAT (A), PLA (B), and PHBH(C).

Table S2. Statistical comparisons of PFOS responses across exposure conditions. Different uppercase letters indicate significant differences among PFOS types within the same exposure time and the same BMPs (Tukey's HSD test, $p < 0.05$). Different lowercase letters indicate significant differences among exposure time within the same PFAS type (Tukey's HSD test, $p < 0.05$).

		**Exposure time**			
**BMPs types**	**PFOS types**	**0.3 days**	**2 days**	**14 days**	**42 days**
**PBAT**	PFBA	C b	C a	C b	D b
	PFOA	C, b	C b	C b	C a
	FOSA	B c	A b	A ab	A a
	PFOS	A b	B ab	B ab	B a
**PBAT-I 10 %**	PFBA	B a	B ab	C ab	D b
	PFOA	B b	B b	C b	C a
	FOSA	A c	A bc	A a	A ab
	PFOS	B c	B bc	B b	B a
**PLA**	PFBA	B -	C -	B -	B -
	PFOA	B a	C a	B a	B a
	FOSA	A a	A b	B b	B b
	PFOS	B bc	B c	A b	A a
**PLA-5 % OPP**	PFBA	B a	A a	B a	B a
	PFOA	C a	B a	C a	B a
	FOSA	A a	B b	C b	B b
	PFOS	C c	B c	A b	A a
**PHBH**	PFBA	B a	B a	B b	B b
	PFOA	C -	C -	B -	B -
	FOSA	A a	A b	B c	B c
	PFOS	B bc	C c	A a	A b
**PHBH-PR10 %**	PFBA	B b	- b	B b	B a
	PFOA	B ab	- b	B a	B b
	FOSA	B -	- -	B -	B -
	PFOS	A b	- c	A b	A a

Table S3. Statistical comparisons of PFAS adsorption across BMPs types at each exposure time. Different lowercase letters indicate significant differences among plastic types within the same PFAS and the same day (Tukey's HSD test, $p < 0.05$).

		BMPs types					
**PFOS Type**	**Times**	**PBAT-P**	**PBAT-I 10 %**	**PLA-P**	**PLA-5 % OPP**	**PHBH-P**	**PHBH-PR10 %**
**PFBA**	**0.3 days**	b	b	b	a	a	b
	**2 days**	b	bc	c	a	a	c
	**14 days**	b	b	b	a	b	b
	**42 days**	b	b	b	a	b	b
**PFOA**	**0.3 days**	b	b	b	b	b	a
	**2 days**	b	a	b	b	b	b
	**14 days**	b	b	b	b	b	a
	**42 days**	a	a	b	b	b	b
**PFOS**	**0.3 days**	ab	c	bc	c	c	a
	**2 days**	a	b	b	b	b	b
	**14 days**	abc	c	c	bc	ab	a
	**42 days**	b	b	b	ab	c	a
**FOSA**	**0.3 days**	c	a	b	b	b	d
	**2 days**	b	a	d	e	c	e
	**14 days**	b	a	c	c	c	c
	**42 days**	a	a	b	b	b	b
